# Standardized survival probabilities and contrasts between hierarchical units in multilevel survival models

**DOI:** 10.1186/s12874-026-02782-8

**Published:** 2026-02-04

**Authors:** Alessandro Gasparini, Michael J. Crowther, Justin M. Schaffer

**Affiliations:** 1 Red Door Analytics AB, Stockholm, Sweden; 2 Department of Cardiothoracic Surgery, Baylor Scott & White The Heart Hospital, Plano, Texas USA

**Keywords:** Multilevel models, Survival analysis, Random effects, Profiling, Risk adjustment

## Abstract

**Background:**

In the medical literature, in which time-to-event (such as time to death or disease recurrence) outcomes are commonly studied, hierarchical data is frequently encountered with patients nested within hospitals or regions. Multilevel hierarchical mixed-effects survival models are routinely used in these settings to accommodate the correlation between study subjects belonging to the same cluster and any potential unobserved heterogeneity. However, these analyses usually focus on fixed effects while marginalizing over the random effects, with fully conditional or marginal (on the random effects) post-estimation predictions.

**Methods:**

In this work, we combine regression standardization over the observed covariates with posterior predictions of the random effects to obtain standardized survival probabilities. These predictions quantify how the entire study population would have fared under the performance of each cluster and can be used to obtain fair comparisons between hierarchical units. Compared to other common approaches, such as the median hazard ratio, this proposal yields quantities that are easier to interpret and that, under certain assumptions, can have a causal interpretation.

**Results:**

We illustrate the new methodology in practice using data on bladder cancer patients with a three-level hierarchical structure composed of patients nested within surgeons and centers. Then, we illustrate a variety of standardized survival predictions benchmarking, e.g., best/average/worst surgeons and centers, surgeons within a center, or centers directly — all from a single and unified modeling framework.

**Conclusions:**

We introduced an analytical approach that can be used to quantify and fairly compare the differences between hierarchical units using easily interpretable measures such as (standardized) survival probabilities and contrasts thereof.

**Supplementary Information:**

The online version contains supplementary material available at 10.1186/s12874-026-02782-8.

## Background

Observational data collected in the human and biological sciences routinely exhibits a hierarchical (or clustered) structure. For instance, siblings tend to be more alike than individuals chosen at random from the population at large, or subjects can be nested within geographical areas or institutions such as schools and hospitals. Multilevel structures arise in longitudinal studies as well, where repeated observations are nested within a given study participant.

Study subjects that are nested (i.e., clustered) within the same hierarchical unit are likely correlated with each other: in these settings, ignoring the multilevel structure can lead to biased and inefficient results [1–5]. Methods that can accommodate this correlation have grown in popularity in recent years: specifically, multilevel, hierarchical models have emerged as a popular analytical tool, given that they allow the simultaneous examination of group-level and individual-level factors [6].

Multilevel models, also known as hierarchical models or mixed-effects models, provide a powerful statistical tool that can be used to analyze data exhibiting hierarchical (or nested) structures by accommodating observed and unobserved covariates, denoted as fixed and random effects, respectively. These models allow for the incorporation of multiple levels of variation within a single and unified analysis and are widely applied in various fields, including psychology, education, sociology, epidemiology, and economics [7–11]. Mixed-effects models can accommodate, among others, continuous, count, and binary outcomes, with increasing levels of complexity [12, 13]. Besides that, multilevel time-to-event (survival) data is frequently encountered in medical research, e.g., studying the survival benefit of heart transplant in patients nested within transplant centers or studying the association between survival and emergent colectomy in colon cancer patients nested within hospitals [14–16].

Traditionally, hierarchical, multilevel models focus on estimating the effect of observed covariates while accounting for the unobserved heterogeneity; in this setting, survival probabilities that marginalize over the random effects have been widely used, with a population-level interpretation. Comparisons between hierarchical units are however crucial in certain situations, for instance, in the process of benchmarking the performance of medical institutions and providers while accounting for differences in the distribution of case-mix covariates (i.e., the covariates identifying each cluster of study subjects) [17, 18]. Measures such as the estimated variance of the random effects or the median hazard ratio have been proposed to quantify the general contextual effect of a given hierarchical level [19–21]; while useful and easy to compute, we argue that these measures lack a clear clinical interpretation., e.g., as they depend on the underlying time-scale and do not directly translate to an absolute risk quantification.

Thus, we propose combining regression standardization with cluster-specific posterior predictions of the random effects to quantify the performance of each hierarchical unit. By fixing the predicted random effects and standardizing over the remaining observed covariates, we obtain model-based predictions that can be compared fairly and that retain the usual interpretation as survival probabilities, either at a specific time point or over the entire observed follow-up time; with multiple hierarchical levels, we can also isolate the effect of a certain level while marginalizing over the remaining ones. Contrasts of standardized survival probabilities can then be computed and have the natural interpretation of risk differences.

Sjölander [22] showed that when covariate adjustment is sufficient to control for confounding, and under usual causal inference assumptions, the above-mentioned contrasts of standardized survival probabilities can have a causal interpretation. In the settings of multilevel models, studies traditionally focused on estimating the causal effect of observed covariates (such as a certain treatment or exposure) while marginalizing over the multilevel structure. In studies with a longitudinal outcome, this is often accomplished by using marginal structural models [23, 24]; nonetheless, fixed-effects models and approaches based on marginalizing over the random effects are popular [25–27]. With this work we propose focusing on the random effects while standardizing over the fixed effects, effectively swapping the target of inference.

This manuscript is organized as follows. We first introduce the applied example that motivated this work. Second, we introduce notation and regression models for survival analysis, the extension to hierarchical settings, and methodology for posterior predictions of the random effects. Then, we introduce regression standardization and frame the proposed approach within the potential outcomes framework, including estimands of interest (and contrasts thereof). In the Estimation section we introduce estimators for the quantities of interest. In the Results section we illustrate the methodology in practice using a dataset of bladder cancer patients. Finally, we discuss the implications of this work in the Discussion section and summarise the main conclusions in the Conclusions section.

### Motivating example

Coronary artery bypass grafting (CABG) is the most common cardiac surgery procedure worldwide [28]. Variation in outcomes between surgeons and hospitals is an area of active study, with both the Centers for Medicare and Medicaid Services (CMS) and the Society of Thoracic Surgeons (STS) now adopting the public reporting of hospital outcomes [29, 30].

Observational data for our ongoing studies, extracted from the CMS administrative claims database, identified over a 1,000,000 CABG patients, treated by 4,000-5,000 surgeons identified with an active practice from 1999-2019 at 1,300-1,500 hospitals across the country. Approaches based on using indicator variables for each surgeon and hospital were avoided as they are likely to overfit, behave erratically, and experience convergence issues [31], especially with such a large sample size and number of nested hierarchical units. Penalized approaches (e.g., using Firth’s correction [32]) were entertained, but we decided to focus on hierarchical models with mixed effects to explicitly model the correlation between units within a cluster [17]. Differences between the approaches are discussed in more detail later.

The CMS data use agreement prevents us from sharing data from the motivating example due to confidentiality and data protection issues. Instead, we illustrate the methodology in practice using a synthetic dataset with three levels of nesting. This dataset is informed by a publicly available dataset with bladder cancer patients nested within 21 centers that participated in the EORTC trial, on top of which we add another layer of nesting with surgeons within the centers. The bladder cancer dataset is included in the {frailtyHL} R package [33, 34], and both datasets can be downloaded from the following GitHub repository, alongside all code that was used to generate the synthetic dataset: https://github.com/RedDoorAnalytics/multilevel-survival-regstd.

## Methods

### Survival analysis notation

Let $T^*$ denote the non-negative random variable for the survival time and $C$ the non-negative random variable for the corresponding censoring time. The observed survival time is defined by the random variable $T = \min (T^*, C)$, and the indicator variable $D = I(T^* \le C)$ denotes either the occurrence of the event of interest or censoring. In practice, each i^th^ study subject yields the bivariate response variable denoted by $(t_i, d_i)$, realizations of $T$ and $D$, respectively.

The survival function $S(t)$ is defined as the complement of the cumulative distribution function $S(t) = 1 - F(t) = 1 - P(T \le t) = P(T > t)$, with corresponding hazard and density functions ($\lambda (t)$ and $f(t)$, respectively) defined as described, among others, by Collett [35]. Regression models for time-to-event data that allow incorporating covariates in any of the quantities introduced above can be defined as well. For instance, commonly used model formulations are the accelerated failure time (AFT) or proportional hazards parametrization (PH); semi-parametric, parametric, and flexible parametric alternatives exist [36–40].

### Hierarchical survival modeling

We now extend the survival modeling framework to the settings of hierarchical data. Specifically, we focus here on a scenario with three levels of nesting, e.g., patients nested within surgeons, with surgeons nested within hospitals; nevertheless, our notation can be easily generalized to any number $l > 1$ levels of nesting. From now onwards, we denote with $Z$ the level-two nesting variable (e.g., the surgeons) and with $H$ the level-three nesting variable (e.g., the hospitals); we will refer to hospitals and surgeons throughout for ease of exposition. We index the subjects with $i = 1, \dots , I_{jk}$, the surgeons with $j = 1, \dots , J_k$, and the hospitals with $k = 1, \dots , K$; $I_{jk}$ denotes the number of patients treated by the j^th^ surgeon at the k^th^ hospital, $J_k$ denotes the number of surgeons at the k^th^ hospital, and $K$ denotes the number of hospitals. Moreover, we define the total study sample size with $n = \sum \nolimits _{k = 1}^K \sum \nolimits _{j = 1}^{J_k} I_{jk}$. From now onwards we focus on survival models in the PH metric, for simplicity, but the methodology directly translates to any model that can be used to predict survival probabilities as well (such as mixed effects accelerated failure time survival models).

A three-level hierarchical PH model parametrizes the hazard function at time $t$ for the i^th^ patient, treated by the j^th^ surgeon in the k^th^ hospital, as

1 $$\begin{aligned} & \lambda _{ijk}(t | X_{ijk}, Z = z_{jk}, H = h_k; \theta , \beta )\nonumber \\ & = \lambda _0(t | \theta ) \exp (X_{ijk} \beta + z_{jk} \alpha _{jk} + h_k \gamma _k) \end{aligned}$$

where $z_{jk}$ is the indicator variable identifying the j^th^ surgeon in the k^th^ hospital (i.e., $I(Z = z_{jk})$), and $h_k$ is the indicator variable identifying the k^th^ hospital (i.e., $I(H = h_k)$). This notation will be useful later on. Moreover, $\beta$ denotes regression coefficients for the fixed effects, $\theta$ denotes ancillary parameters for the baseline hazard function, and $\alpha _{jk}$ and $\gamma _k$ denote the random intercepts for the latent effect of the j^th^ surgeon in the k^th^ hospital and the k^th^ hospital, respectively. We assume normally distributed random effects on the surgeon and hospital effects, with $\alpha _{jk} \sim N(0, \sigma ^2_{\alpha })$ and $\gamma _k \sim N(0, \sigma ^2_{\gamma })$. The covariance between the surgeon- and hospital-level random effects is zero due to assuming nested random effects, which occurs by design in the settings or hierarchical models. Note that we can reformulate the hierarchical model of Eq. 1 in terms of survival probabilities.

As outlined by Varewyck et al. [17] and Chen et al. [18], hierarchical models can be used in the context of profiling providers’ performance to estimate the cluster effects while adjusting for a set of case-mix factors $X$. Specifically, the performance (or quality) of a hierarchical unit can be quantified by calculating posterior predictions of the random effects $\alpha _{jk}$ and $\gamma _k$; in the settings of survival models, this is commonly done by using empirical Bayes means and modes (also known as posterior means and modes). These are introduced in the next section.

Varewyck et al. [17] compare the random effects approach with a fixed effects approach (where indicator variables for each center and surgeon are included as fixed effects) and with a penalized fixed effects approach (using Firth’s correction [32]). While random effects models did introduce some bias in the estimation of the variance components when the number of clusters was small, they can stabilize the prediction of the random effects by shrinking their value to the population average; the amount of shrinkage is inversely proportional to the amount of data in each cluster [17, 41]. On the other side, fixed effect models can lead to computational issues and unstable estimation given the (possibly very) large number of parameters to be estimated [31]; for instance, in our motivating dataset, parametrizing each hospital and surgeon with a fixed effect would require several thousand parameters. This would make alternative methods, such as that introduced by Van Rompaye et al. [42], unfeasible. Another alternative is given by marginal modeling with the correlation between subjects in a certain unit modeled via the variance-covariance matrix [43]. Nonetheless, the random effects approach has important advantages: it directly yields an estimate of the heterogeneity between hierarchical units, it helps reduce the effective model dimension, it allows more flexibility as both conditional and marginal predictions can be obtained post-estimation, and yields higher specificity and positive predictive value when classifying outliers (at the cost of lower sensitivity, with small sample sizes) [44, 45].

Model-based predictions from the survival models described in this section can be used to calculate directly standardized estimates of hospital/surgeon-specific outcomes, as outlined in the following sections.

### Posterior predictions of the random effects

Empirical Bayes predictors of the random effects are the means (or modes) of their empirical posterior distribution with model parameters $\theta , \beta$ (for the PH model of Eq. 1) replaced with their estimated values or $\hat{\theta }, \hat{\beta }$. We denote empirical Bayes predictions of the random effects with $\tilde{\alpha }_{jk}$ and $\tilde{\gamma }_k$ to distinguish predicted values from estimates; some argue, however, that this distinction is rather subtle [46].

Focusing on a general two-level model for ease of exposition, the empirical posterior distribution of a random intercept $u_i$ (for a certain i^th^ cluster), conditional on observed data $y$, fixed-effects $X$, random effects $Z$, estimated model parameters $\hat{\theta }$ and $\hat{\beta }$, is:

$$\begin{aligned} \omega (u_i | y_i, X_i, Z_i; \hat{\theta }, \hat{\beta }, \hat{\Sigma }) = \frac{f(y_i | u_i, X_i, Z_i; \hat{\theta }, \hat{\beta }) \phi (u_i; \hat{\Sigma })}{L_i(\hat{\theta }, \hat{\beta })} \end{aligned}$$

where $f(\cdot )$ is the conditional density function for the response variable, $\phi (\cdot )$ is the density function of a normal distribution with mean zero and variance-covariance matrix $\hat{\Sigma }$ (the estimated variance-covariance matrix of the random effects), and $L_i(\cdot )$ is the likelihood contribution of the i^th^ cluster (described in more detail elsewhere [47]). Empirical Bayes mean predictions of the random effects can then be calculated as:

$$\begin{aligned} \tilde{u}_i = \int u_i \omega (u_i | y_i, X_i, Z_i; \hat{\theta }, \hat{\beta }, \hat{\Sigma }) d u_i \end{aligned}$$

Note that the integral above does not have a closed form and needs to be approximated, e.g., using numerical integration. Conversely, empirical Bayes mode predictions are approximated by solving the following equation for the mode $\tilde{\tilde{u}}_i$:

$$\begin{aligned} \frac{\partial }{\partial u_i} \log \omega (\tilde{\tilde{u}}_i | y_i, X_i, Z_i; \hat{\theta }, \hat{\beta }, \hat{\Sigma }) = 0 \end{aligned}$$

In time-to-event and generalized mixed-effects models, the posterior density $\omega (\cdot )$ tends to a multivariate normal as cluster size increases; moreover, as the posterior density approaches a multivariate normal distribution, empirical Bayes means and modes get closer to each other. This generalizes beyond two-level models by applying orthogonalizing transformations using the Cholesky transformation, as the random effects are no longer independent under the posterior distribution. For this manuscript, we focus on empirical Bayes means as this approach minimizes the posterior mean-squared error of prediction, given outcome values and covariates; conversely, an advantage of posterior modes is that they can be computationally more efficient to calculate since they do not require numerical integration. More details on the comparison between posterior means and modes are included in Skrondal and Rabe-Hesketh [48].

Finally, standard errors for the posterior predictions of the random effects can be estimated using the posterior standard deviation (for empirical Bayes means) or using standard maximum likelihood theory (for empirical Bayes modes); a review of different methods for calculating standard errors of predicted random effects is in Skrondal and Rabe-Hesketh [48].

Note that in the work of Varewyck et al. [17], where they rely on linear mixed-effects models, empirical Bayes means correspond to the usual best linear unbiased predictions (BLUPs) of the random effects.

### Standardized survival probabilities

The outcome of interest for our study is time to the occurrence of a certain event. Effects in these settings have been traditionally quantified using the hazard ratio; however, this is not feasible in our settings as we would be comparing the predicted random effect for each cluster versus a reference hypothetical cluster (with a random effect of zero). Moreover, this commonly reported measure suffers from non-collapsibility: it is well known that, even in the ideal settings of a perfect randomized controlled clinical trial (RCT), a hazard ratio will change upon including a baseline covariate in the model whenever that covariate is associated with the outcome [49–51]. In other words, even if there is no confounding, the inclusion of a covariate in a model matters for the magnitude of the treatment effect: conditioning on a covariate changes the very nature of the study estimand. AFT models overcome this limitation, as illustrated by Crowther et al. [40], and are thus often recommended when collapsible estimands are of interest; this is further discussed, among others, by Martinussen and Vansteelandt [52], Sjölander et al. [50], and Daniel et al. [51]. Here we take an alternative approach and focus on survival probabilities instead, which have a natural interpretation close to that of risks.

Several types of model-based survival probability predictions can be obtained after fitting a hierarchical survival model. Fully conditional model-based predictions require fixing a certain value for each covariate (fixed effect) included in the model plus posterior predictions of the random effects and can be interpreted as the predicted survival probability at a certain time $t$ for a subject in a specific cluster and with a specific covariates profile. These quantities are, however, inappropriate to compare hierarchical units (surgeons, hospitals) as they do not capture the entire range of subjects within a cluster. Thus, we propose using regression standardization to average over a fixed covariates distribution (case mix) while fixing the predicted random effects for a certain surgeon and hospital combination.

Formally, the standardized survival probability $S_s$ over the case-mix covariates $X$ assuming the effect of surgeon $Z = z_{jk}$ from hospital $H = h_k$, at time $t$, can be defined as

2 $$\begin{aligned} S_s(t | Z = z_{jk}, H = h_k) = E[S(t | Z = z_{jk}, H = h_k, X)], \end{aligned}$$

with the expectation taken over $X$. The case-mix $X$ usually refers to the entire study population, but it may denote the case-mix of specific clusters or subgroups of the study population as well, depending on the study aims.

This quantity is akin to direct standardization and can be interpreted as the survival probability at time $t$ for the entire study population under the performance of surgeon $Z = z_{jk}$ and hospital $H = h_k$. Most literature on profiling health care centers uses indirect standardization instead, which compares the observed outcome in a certain center versus “what would have been if the average performance of surgeons and hospitals applied” [17, 53, 54]; in other words, indirect standardization would evaluate each center on its own case-mix and compare it to how an average center would perform on the same patients. This is most relevant for policymakers, but direct standardization is more interesting when centers are expected to perform well on the entire study population, as in our settings.

In the following section, we borrow from the causal inference literature to expand on and formally define the different kinds of standardized survival probability predictions that could be calculated after fitting hierarchical survival models.

### Standardized survival probabilities within the potential outcomes framework

We previously introduced standardized survival probabilities and motivated their use in the settings of this study. We now formally define all potential quantities of interest and frame these within the potential outcomes framework.

In the traditional settings of hierarchical survival analysis, interest is often in contrasts involving fixed effects coefficients (e.g., any of the case-mix variables $X_{ijk}$), and the random effects are treated as a nuisance. Specifically, say we are interested in estimating the counterfactual survival probability for a hypothetical subject with a certain level of a covariate $X^+_{ijk}$, where $X_{ijk}$ is partitioned into $(X^+_{ijk}, X^*_{ijk})$. This leads to estimands such as those introduced in Eqs. 3 and 4:

3 $$\begin{aligned} & S^{X^+ = x, Z = z_{jk}, H = h_k}(t)\nonumber \\ & = E[S(t | X^+ = x, X^*, Z = z_{jk}, H = h_k)] \end{aligned}$$

4 $$\begin{aligned} S^{X^+ = x}(t) = E[S(t | X^+ = x, X^*, Z, H)] \end{aligned}$$

Equation 3 fixes the level of a certain case-mix covariate $X^+ = x$ and the surgeon and hospital effect and can be interpreted as the counterfactual survival probability for subjects with that level of $X^+$, treated by the j^th^ surgeon at the k^th^ hospital, while averaging over every other case-mix covariate $X^*$. Analogously, Eq. 4 marginalizes over surgeons and hospitals as well: this can be interpreted as the marginal survival probability, in the entire study population of interest, for subjects with a certain level of the exposure covariate $X^+ = x$.

**Fig. 1:**
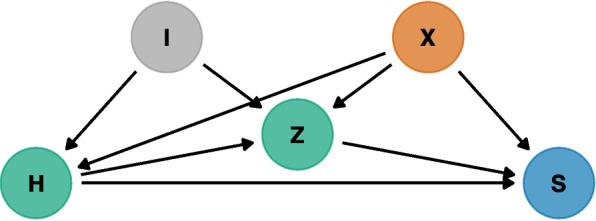
Simplified directed acyclic graph denoting the assumed causal structure. $S$ denotes the outcome of interest, $X$ denotes confounders for the case-mix adjustment, $H$ denotes the hospital assignment, $Z$ denotes the surgeon assignment, and $I$ denotes a set of variables that could influence the hospital/surgeon assignment

These equations are introduced for completeness but are not used in this work. Instead, we focus on studying the effect of surgeons and hospitals on the outcome and treat the case-mix variables as a nuisance that we adjust/account for to obtain a fair comparison and quantification of the performance of higher-level units. The causal structure that we assume for this problem is included in the directed acyclic diagram (DAG) of Fig. 1; there, $S$ denotes the outcome of interest, $X$ denotes confounders to use for the case-mix adjustment, $H$ denotes the hospital assignment, $Z$ denotes the surgeon assignment, and $I$ denotes a set of variables that could influence the hospital/surgeon assignment (but are independent of case-mix covariates $X$). We do not include potential unmeasured confounders in the DAG, but that is something to still be aware of.

Formally, we denote the potential outcome (survival probability) $S$ at time $t$ for a subject treated by the j^th^ surgeon in the k^th^ hospital with $S^{Z = z_{jk}, H = h_k}(t)$. Further, we denote the potential outcome for a subject treated by the j^th^ surgeon (at the k^th^ hospital) with $S^{Z = z_{jk}}(t)$ and the potential outcome for a subject treated at the k^th^ hospital with $S^{H = h_k}(t)$. The first quantity denotes the case-mix adjusted counterfactual survival probability, at time $t$, for a given (hospital, surgeon) combination. The second and third quantities denote the case-mix adjusted counterfactual survival probability for the j^th^ surgeon (marginally over hospitals) and for the k^th^ hospital (marginally over surgeons). Note that these three key quantities are all considered marginally over the case-mix covariates.

Mathematically, we can define the first quantity as

5 $$\begin{aligned} S^{Z = z_{jk}, H = h_k}(t) = E[S(t | X, Z = z_{jk}, H = h_k)] \end{aligned}$$

with the expectation in Eq. 5 taken over the distribution of the fixed effects, case-mix variables $X$. Note that we fix the surgeon effect to that of surgeon $Z = z_{jk}$ and the hospital effect to that of hospital $H = h_k$.

Analogously, we define the second and third quantities as:

6 $$\begin{aligned} S^{Z = z_{jk}}(t) = E[S(t | X, Z = z_{jk}, H)] \end{aligned}$$

7 $$\begin{aligned} S^{H = h_k}(t) = E[S(t | X, Z, H = h_k)] \end{aligned}$$

where the expectations are taken over the distribution of the fixed effects $X$ and the random hospital effect in Eq. 6, fixed effects $X$, and random surgeon effect in Eq. 7. Estimators for these three quantities are introduced in the Estimation section.

The quantities discussed in this section are often referred to as the result of direct standardization and can be interpreted as the risk that would be realized if the entire study population was exposed to the care level of the j^th^ surgeon and at the k^th^ hospital (for Eq.5). Analogously, Eq. 6 can be interpreted as the risk that would be realized if the entire study population was exposed to the care level of the j^th^ surgeon, and Eq. 7 as the risk if the entire study population was exposed to the care level of the k^th^ hospital, marginally over everything else (case-mix and hospitals/surgeons). In these settings the case-mix used as a reference is a common set of subjects across all clusters, and as such, between-cluster comparisons are based on their performance in the extended patient population. There is of course a risk for extrapolation here, as this approach may evaluate a cluster’s performance based on patients that it is not likely to treat: this issue needs to be assessed on a case-by-case basis, to minimize such risk.

Chen et al. [18] highlight that causal inference on the hospital and surgeon effects is possible under certain assumptions. First, the observed outcomes are linked to their potential counterparts under the counterfactual consistency and stable unit treatment value assumptions. Second, a required assumption is that of strong ignorability of the joint hospital and surgeon assignment mechanism, which states that $0< P(Z = z_{jk}, H = h_k | X_{ijk}) < 1$ for all $i, j, k$ (positivity) and that $S^{Z = z_{jk}, H = h_k}(t) {\perp \!\!\!\perp } (Z, H) | X$ (conditional exchangeability). Positivity implies that every study subject could (in principle) be treated by any surgeon and hospital. Third, one needs to assume exchangeability within clusters, i.e., that conditionally on the observed case-mix covariates and random effects, individuals within the same cluster are exchangeable. Finally, one needs to assume that the measured case-mix covariates are sufficient for confounding control (i.e., no leftover unmeasured confounding), that censoring is not informative, and that the hierarchical survival model is correctly specified.

#### Contrasts

We can combine the estimands defined in the previous section to derive relevant contrasts involving those quantities. Sjölander [22] remarks that if adjusting for the case-mix variables is sufficient to control confounding, then the resulting contrasts of standardized survival probabilities have a causal interpretation as a risk difference.

Specifically, we define as a contrast of interest the difference in case-mix adjusted survival probabilities between two (hospital, surgeon) combinations:

8 $$\begin{aligned} & \eta ^{(Z = z_{j^1 k^1}, H = h_{k^1}),(Z = z_{j^0 k^0}, H = h_{k^0})}(t)\nonumber \\ & = S^{Z = z_{j^1 k^1}, H = h_{k^1}}(t) - S^{Z = z_{j^0 k^0}, H = h_{k^0}}(t) \end{aligned}$$

In Eq. 8 we contrast the case-mix adjusted survival probability between two distinct hospital and surgeon combinations. This can be interpreted as the difference in case-mix adjusted survival probability at $t$ years when being treated by surgeon $Z = z_{j^1 k^1}$ at hospital $H = h_{k^1}$ versus being treated by surgeon $Z = z_{j^0 k^0}$ at hospital $H = h_{k^0}$.

Of course, contrasts can be defined conditionally on a given hospital or surgeon. For instance, contrasting surgeons $Z = z_{j^1}$ vs $Z = z_{j^0}$ within a given hospital $H = h_{k}$ leads to the following contrast:

9 $$\begin{aligned} & \eta ^{(Z = z_{j^1 k}, H = h_{k}),(Z = z_{j^0 k}, H = h_{k})}(t) \nonumber \\ & = S^{Z = z_{j^1 k}, H = h_{k}}(t) - S^{Z = z_{j^0 k}, H = h_{k}}(t) \end{aligned}$$

Equation 9 denotes the survival difference between two surgeons for a fixed hospital effect, marginally over the case-mix covariates. Note that the above example is only provided for illustration purposes: in these settings, it is key to define realistic contrasts that correspond to plausible interventions [55].

The contrast defined in Eq. 9 can be thought of as a contrast of surgeon effects, conditionally on a certain hospital effect but marginally over the case-mix covariates. We can extend this to use the survival probabilities defined in Eqs. 6 and 7 instead to define contrasts that are marginal over either hospitals or surgeons:

10 $$\begin{aligned} \eta ^{(Z = z_{j^1 k}),(Z = z_{j^0 k})}(t) = S^{Z = z_{j^1 k}}(t) - S^{Z = z_{j^0 k}}(t) \end{aligned}$$

The contrast in Eq. 10 defines a contrast between surgeons $j^1$ and $j^0$, marginally over the case-mix variables and the hospital effects. The contrast introduced in Eq. 10 is thus net of the effect of case-mix variables and hospitals, given standardization over those factor; once again, care is required to ensure that contrasts of this kind are realistic. Analogously, marginal contrasts of hospital effects can be defined, e.g., comparing hospitals $k^1$ and $k^0$:

11 $$\begin{aligned} \eta ^{(H = h_{k^1}),(H = h_{k^0})}(t) = S^{H = h_{k^1}}(t) - S^{H = h_{k^0}}(t) \end{aligned}$$

We summarise each estimand and their interpretation in Table 1. Note that, given the assumed hierarchical model formulation, the contrasts introduced in this section rely on the proportional hazards assumption as well.

**Table 1:** Summary and interpretation of each estimand

Equation	Estimand and Interpretation
Equation 3	$S^{X^+=x, Z = z_{jk}, H = h_k}(t)$: Counterfactual survival probability for a hypothetical subject with a certain level of case-mix variable $X^+ = x$ and treated at the j^th^ hospital by the k^th^ surgeon, while averaging over the remaining case-mix covariates.
Equation 4	$S^{X^+=x}(t)$: Counterfactual survival probability for a hypothetical subject with a certain level of case-mix variable $X^+ = x$ in the entire population of interest, averaging over the remaining case-mix covariates, surgeons, and hospitals.
Equation 5	$S^{Z = z_{jk}, H = h_k}(t)$: Counterfactual survival probability for a subject treated by the j^th^ surgeon at the k^th^ hospital, marginally over the case-mix covariates.
Equation 6	$S^{Z = z_{jk}}(t)$: Counterfactual survival probability for a subject treated by the j^th^ surgeon, marginally over the case-mix covariates and over hospitals.
Equation 7	$S^{H = h_k}(t)$: Counterfactual survival probability for a subject treated at the k^th^ hospital, marginally over the case-mix covariates and over surgeons.
Equation 8	$\eta ^{(Z = z_{j^1 k^1}, H = h_{k^1}),(Z = z_{j^0 k^0}, H = h_{k^0})}(t)$: Contrast (difference) of counterfactual survival probabilities for a subject treated by surgeon j^1^ at hospital k^1^ versus surgeon j^0^ at hospital k^0^, marginally over the case-mix covariates.
Equation 9	$\eta ^{(Z = z_{j^1 k}, H = h_{k}),(Z = z_{j^0 k}, H = h_{k})}(t)$: Contrast (difference) of counterfactual survival probabilities for a subject treated at the k^th^ hospital by surgeon j^1^ versus surgeon j^0^, at the same hospital, marginally over the case-mix covariates. Analogous contrasts can be defined by swapping surgeons and hospitals in Eq. 9 and fixing the surgeon effect instead.
Equation 10	$\eta ^{(Z = z_{j^1 k}),(Z = z_{j^0 k})}(t)$: Contrast (difference) of counterfactual survival probabilities for a hypothetical subject treated by the surgeon j^1^ versus surgeon j^0^, marginally over the case-mix covariates and the hospitals.
Equation 11	$\eta ^{(H = h_{k^1}),(H = h_{k^0})}(t)$: Contrast (difference) of counterfactual survival probabilities for a hypothetical subject treated at hospital k^1^ versus hospital k^0^ instead, marginally over the case-mix covariates and the surgeons.

### Estimation

#### Survival probabilities

The estimation procedure for any of the quantities described in the previous section starts by fitting a hierarchical survival model. Focusing again on PH models for simplicity (with alternative models and approaches of course possible), we obtain estimated model parameters $\hat{\theta }, \hat{\beta }$ and estimated variances of each random effect $\hat{\sigma }^2_{\alpha }, \hat{\sigma }^2_{\gamma }$, which we can use to obtain posterior predictions of the random effects $\tilde{\alpha }_{jk}, \tilde{\gamma }_k$ for any $j, k$. These values are plugged in the survival function for a hierarchical model (such as that of Eq. 1) to obtain the fully conditional survival probability for a subject with case-mix profile $X_{ijk}$, treated by the j^th^ surgeon at the k^th^ hospital:

12 $$\begin{aligned} & \hat{S}_{ijk}(t | X_{ijk}, Z = z_{jk}, H = h_k; \hat{\theta }, \hat{\beta })\nonumber \\ & = S_0(t | \hat{\theta })^{\exp (X_{ijk} \hat{\beta } + \tilde{\alpha }_{jk} + \tilde{\gamma }_k)} \end{aligned}$$

Estimators for all the quantities introduced in the previous section (and summarised in Table 1) can be defined using regression standardization [56–59]. Here we focus on quantities that treat the case-mix variables as nuisance: estimators for other quantities (such as those in Eqs. 3 and 4) are defined elsewhere, e.g., by Dahlqwist et al. [26].

First, we can define the case-mix adjusted counterfactual survival probability at time $t$ for a given (hospital, surgeon) combination ($Z = z_{j^*k^*}, H = h_{k^*}$, Eq. 5):

13 $$\begin{aligned} & \hat{S}^{Z = z_{j^* k^*}, H = h_{k^*}}(t)\nonumber \\ & = \frac{1}{n} \sum \limits _{k = 1}^K \sum \limits _{j = 1}^{J_k} \sum \limits _{i = 1}^{I_{jk}} \hat{S}(t | X_{ijk}, Z = z_{j^*k^*}, H = h_{k^*}; \hat{\theta }, \hat{\beta }) \nonumber \\ & = \frac{1}{n} \sum \limits _{k = 1}^K \sum \limits _{j = 1}^{J_k} \sum \limits _{i = 1}^{I_{jk}} S_0(t | \hat{\theta })^{\exp (X_{ijk} \hat{\beta } + \tilde{\alpha }_{j^*k^*} + \tilde{\gamma }_{k^*})} \end{aligned}$$

where $\hat{S}(\cdot )$ follows from Eq. 12 (if we used a hierarchical survival model in the PH metric) or from any equivalent formula, and $n$ denotes the overall sample size of the study. Note that in Eq. 13 we plug in the predicted random effects for surgeon $j^*$ in hospital $k^*$, effectively imposing the effect of that specific surgeon and hospital combination on the entire study population while marginalizing over the observed distribution of the fixed effects.

For the case-mix adjusted counterfactual survival probability at time $t$ for a given surgeon $j^*$, marginally over fixed effects and hospitals (Eq. 6), we can define the following estimator:

14 $$\begin{aligned} & \hat{S}^{Z = z_{j^* k^*}}(t)\nonumber \\ & = \frac{1}{n} \sum \limits _{k = 1}^K \sum \limits _{j = 1}^{J_k} \sum \limits _{i = 1}^{I_{jk}} \hat{S}(t | X_{ijk}, Z = z_{j^*k^*}, H; \hat{\theta }, \hat{\beta }) \nonumber \\ & = \frac{1}{n} \sum \limits _{k = 1}^K \sum \limits _{j = 1}^{J_k} \sum \limits _{i = 1}^{I_{jk}} \int _{-\infty }^{+\infty } S_0(t | \hat{\theta })^{\exp (X_{ijk} \hat{\beta } + \tilde{\alpha }_{j^*k^*} + g)} \phi _{\gamma }(g; \hat{\sigma }^2_{\gamma }) \ dg \end{aligned}$$

The inner integral marginalizes over the distribution of the hospital-level random effect, $\phi _{\gamma }(g; \sigma ^2_{\gamma }) = N(0, \sigma ^2_{\gamma })$, and does not have a closed form; this can be approximated numerically (e.g., using numerical quadrature). Analogously, the case-mix adjusted counterfactual survival probability at time $t$ for a given hospital $k^*$, marginally over fixed effects and surgeons (Eq. 7), follows from Eq. 14 by swapping hospital and surgeons:

15 $$\begin{aligned} & \hat{S}^{H = h_{k^*}}(t)\nonumber \\ & = \frac{1}{n} \sum \limits _{k = 1}^K \sum \limits _{j = 1}^{J_k} \sum \limits _{i = 1}^{I_{jk}} \hat{S}(t | X_{ijk}, Z, H = h_{k^*}; \hat{\theta }, \hat{\beta }) \nonumber \\ & = \frac{1}{n} \sum \limits _{k = 1}^K \sum \limits _{j = 1}^{J_k} \sum \limits _{i = 1}^{I_{jk}} \int _{-\infty }^{+\infty } S_0(t | \hat{\theta })^{\exp (X_{ijk} \hat{\beta } + a + \tilde{\gamma }_{k^*})} \phi _{\alpha }(a; \hat{\sigma }^2_{\alpha }) \ da \end{aligned}$$

Note that the same considerations discussed above regarding Eq. 14 apply to Eq. 15 as well.

#### Contrasts

All contrasts estimands can be estimated by combining the estimators introduced in the previous section. For instance, the contrast (difference) of counterfactual survival probabilities for a subject treated by surgeon j^1^ at hospital k^1^ versus being treated by surgeon j^0^ at hospital k^0^, marginally over the case-mix covariates, is defined as:

16 $$\begin{aligned} & \hat{\eta }^{(Z = z_{j^1 k^1}, H = h_{k^1}),(Z = z_{j^0 k^0}, H = h_{k^0})}(t)\nonumber \\ & = \hat{S}^{Z = z_{j^1 k^1}, H = h_{k^1}}(t) - \hat{S}^{Z = z_{j^0 k^0}, H = h_{k^0}}(t) = \nonumber \\ & = \frac{1}{n} \sum \limits _{k = 1}^K \sum \limits _{j = 1}^{J_k} \sum \limits _{i = 1}^{I_{jk}} S_0(t | \hat{\theta })^{\exp (X_{ijk} \hat{\beta } + \tilde{\alpha }_{j^1k^1} + \tilde{\gamma }_{k^1})}\nonumber \\ & \quad - \frac{1}{n} \sum \limits _{k = 1}^K \sum \limits _{j = 1}^{J_{k}} \sum \limits _{i = 1}^{I_{jk}} S_0(t | \hat{\theta })^{\exp (X_{ijk} \hat{\beta } + \tilde{\alpha }_{j^0k^0} + \tilde{\gamma }_{k^0})} \end{aligned}$$

Estimators for the other contrasts can be analogously defined by plugging in the relevant quantities and are omitted here for simplicity.

#### Variance

In the previous sections we have only discussed methods for obtaining point estimates and contrasts of case-mix adjusted counterfactual survival probabilities. We now introduce a procedure to estimate standard errors for any of those quantities.

Let $\psi$ be an estimand (or contrast) of interest, with point estimate $\hat{\psi }$, and let the model parameters be normally distributed, $[\theta \ \beta ]^\top \sim \text {MVN} \left( [\hat{\theta } \ \hat{\beta }]^\top , \hat{\Sigma }_{\hat{\theta }, \hat{\beta }}\right)$, with $\hat{\Sigma }_{\hat{\theta }, \hat{\beta }}$ obtained by, e.g., inverting the Hessian matrix at the optimum. We also assume approximate normality of the subject-specific posterior predictions [48]: $\alpha _{jk} \sim N(\tilde{\alpha }_{jk}, \hat{\sigma }^2_{\tilde{\alpha }_{jk}})$ and $\gamma _k \sim N(\tilde{\gamma }_k, \hat{\sigma }^2_{\tilde{\gamma }_k})$, where $\tilde{\alpha }_{jk}$ and $\tilde{\gamma }_k$ denote the cluster-specific posterior predictions for a given surgeon and hospital, which are predicted with standard errors $\hat{\sigma }^2_{\tilde{\alpha }_{jk}}$ and $\hat{\sigma }^2_{\tilde{\gamma }_k}$. Alternatively, one could assume a location-scale t distribution as proposed by Rizopoulos [60].

Then, we can use the following procedure to estimate the variance of any model-based predictions, based on previous work by Chen et al. [61]:

1. Draw new model parameters $[\theta _b, \beta _b]^\top$ from their multivariate normal distribution;
2. Draw new posterior predictions of the random effects $\tilde{\alpha }_{jk, b}$ and $\tilde{\gamma }_{k, b}$ from their distributions;
3. Re-compute the estimand/contrast of interest, $\hat{\psi }_b$, using the new model parameters and random effects from the previous steps;
4. Repeat the above procedure $B$ times to obtain $\hat{\psi }_1, \hat{\psi }_2, \dots , \hat{\psi }_b, \dots , \hat{\psi }_B$.

An empirical estimate of the standard error of $\hat{\psi }$ can then be calculated by taking the square root of the variance across the $B$ repetitions:

$$\begin{aligned} \widehat{\text {Var}}[\hat{\psi }] = \frac{1}{B} \sum \limits _{b = 1}^B [ \hat{\psi }_b - \mu ]^2, \quad \mu = \frac{1}{B} \sum \limits _{b = 1}^B \hat{\psi }_b \end{aligned}$$

The estimated standard error can then be used to construct confidence intervals for the point estimate; alternative methods, such as the percentile method across the $B$ repetitions, could be used as well.

Alternatively, one could use a non-parametric bootstrap procedure based on resampling study subjects with replacement. However, if we sample without considering the multilevel structure of our data, we risk destroying the natural hierarchy of the data: to avoid this issue, one could either resample at the highest level (e.g., the hospitals), if the number of clusters is large, or apply ad-hoc methods such as the hierarchical residuals bootstrap introduced by Carpenter et al. [62]. Note that non-parametric hierarchical bootstrap based on resampling study subjects at the highest level is computationally more challenging, having to re-fit the multilevel survival model with each bootstrap replicate. Moreover, non-parametric approaches based on bootstrapping the residuals instead may not be appropriate, as highlighted by Morris [63] in the settings of linear mixed models.

#### Software

User-friendly implementations of this methodology are provided to enable its use in practice. Specifically, we provide a Stata [64] post-estimation command for the -mestreg- command, named -stdmest-, which is available on GitHub at https://github.com/RedDoorAnalytics/stdmest and can be installed from the Stata console with the net install command. We also provide an R [65] package implementing the post-estimation prediction algorithms, which is also available on GitHub at https://github.com/RedDoorAnalytics/stdmest-r. Note that the R package requires estimating a hierarchical survival model using -mestreg- in Stata first; utility functions to export the model fit (in Stata) and to import it in a correct format (in R) are provided as well. Other software packages for fitting multilevel survival models could be used [66–68], as long as all quantities required to re-implement the estimators can be obtained.

## Results

For this application, we analyze the three-level data introduced in the Motivating example section. An additional analysis of the two-level data is included in the supplementary material available online, for completeness, including a comparison of the two datasets.

We fit a hierarchical survival model assuming center- and surgeon-level random intercepts. Moreover, we assume i) proportional hazards and ii) a Weibull baseline hazard function, while iii) adjusting for age, sex, and use of chemotherapy; these models are estimated using the Stata command -mestreg- and its default settings. Note that these modeling assumptions are for illustration purposes only; in practice, more complex model-building strategies are likely required. We then use the algorithm described in the Variance section with B = 1,000 repetitions and the percentile method to obtain confidence intervals for all post-estimation predictions.

Data and annotated statistical code (in Stata and R) to fully replicate our analyses can be downloaded from the following GitHub repository: https://github.com/RedDoorAnalytics/multilevel-survival-regstd.

The goal of this application is to quantify heterogeneity and differences between centers and surgeons. Traditionally, these have been quantified using either the estimated variance of the random intercept or the median hazard ratio (MHR). In the settings of this application, the estimated variance of the random intercept was 0.018 (95% C.I.: 0.012, 0.026) and 0.019 (0.009, 0.038) at a surgeon and center level, respectively. While this summary measure is straightforward to calculate, its interpretation is not intuitive; for instance, the variance of the random intercept does not translate directly to risk, and its magnitude depends on the underlying timescale (e.g., days, years, etc.). The MHR improves on that, e.g., quantifying that the hazard ratio comparing a high-risk center versus a low-risk center is, at a median level, 1.140. This means that the hazard in high-risk hospitals is approximately 14.0% higher than the hazard in low-risk hospitals (again, at a median level and if we were performing all possible comparisons). Analogously, the estimated MHR for surgeons was 1.135, highlighting that the hazard in high-risk surgeons is approximately 13.5% higher than the hazard in low-risk surgeons. Nonetheless, the MHR is a relative measure that only has an overall, population-level interpretation; we now move beyond that by estimating and presenting standardized survival probabilities for specific centers and surgeons. The following post-estimation predictions are directly comparable as we standardize over the same covariate distribution (i.e., that of the entire study dataset).

**Fig. 2:**
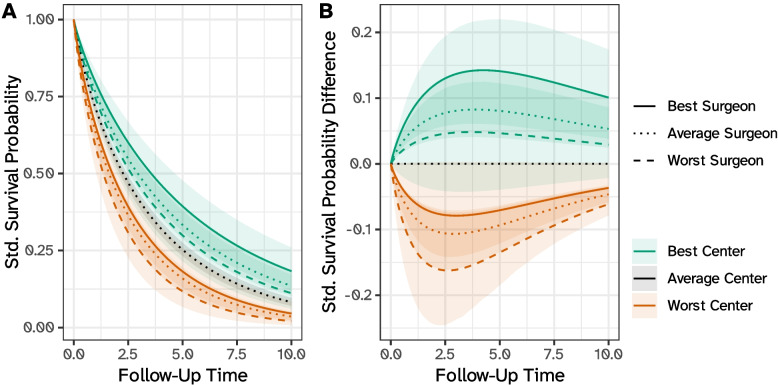
Standardized survival probabilities (panel **A**) and differences (panel **B**) for the best, worst, and (theoretical) average surgeon from the best, worst, and (theoretical) average center, according to the three-level model with patients nested within centers and surgeons. Standardized survival probability differences assume the average surgeon from the average center as the reference

We start by calculating posterior predictions of the random effects for each center and surgeon using the empirical Bayes means approach (see the Posterior predictions of the random effects section); then, we fix posterior predictions at each hierarchical level to obtain predictions and contrasts analogous to those defined in Eqs. 5 and 8. Specifically, say we are interested in comparing the best, worst, and average surgeon from the best, worst, and (theoretical) average center, taking the (theoretical) average surgeon in the average center as the overall reference. Note that the theoretical average hospital (or surgeon) assumes the mean value of the random intercept distribution ($\alpha _{jk} = 0$ or $\gamma _k = 0$), and therefore does not necessarily refer to a real cluster; the best and worst hospital (and surgeon) are identified by the smallest and largest predicted random effect, respectively. Predictions and contrasts are depicted in Fig. 2: from these plots, we can quantify that at, e.g., 10 years the average surgeon in the best center has a standardized survival probability of 0.135 (95% C.I.: 0.104, 0.172) compared to a probability of 0.036 (0.024, 0.051) for the average surgeon in the worst center. This difference is larger (in absolute terms) than, for instance, the difference between the best and worst surgeon in the best center (0.183 vs 0.111) or in the worst center (0.045 vs 0.021), highlighting that differences between centers are likely larger than differences between surgeons.

**Fig. 3:**
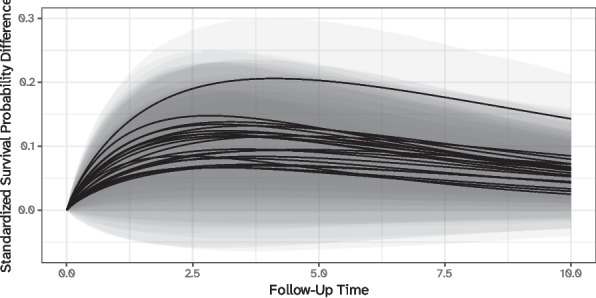
Standardized survival probability differences between the best and worst surgeon in each center. Each line in the plot denotes a distinct center

More detailed contrasts, either focusing on specific combinations of surgeons and centers or across the entire distribution, could be analogously defined and computed. This is explored in more detail in the two-level example in the supplementary material. As an example, a certain study might want to quantify within-center differences, e.g., by comparing the performance of the best and worst surgeon of each center: these standardized survival probability differences are depicted in Fig. 3, where each line identifies a specific center. These predictions highlight that, even after standardizing over study covariates, large differences between surgeons and within centers persist.

**Fig. 4:**
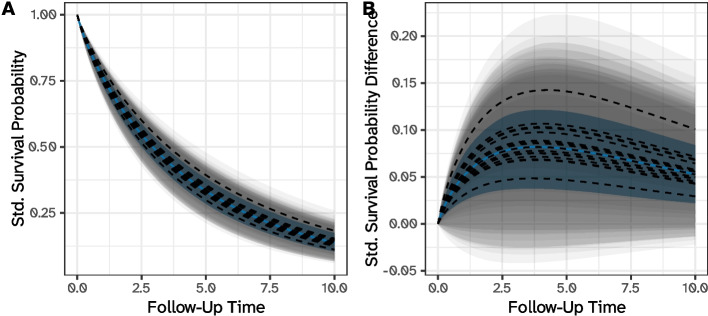
Standardized survival probabilities (panel **A**) and differences (panel **B**) for (1) all surgeons in the best center, in black, and (2) the best center while marginalizing over the surgeons, in blue. Predictions are based on the three-level model with patients nested within centers and surgeons, and differences assume the theoretical average as the reference

As previously discussed, we can also obtain predictions for a certain hierarchical level (e.g., centers) while averaging over another (e.g., surgeons): these are formally defined in Eqs. 6 and 7, with contrasts in Eqs. 11 and 10. To this end, we focus on the best center (e.g., the center with the smallest posterior prediction of the random intercept) and obtain predictions for every surgeon within the center. These predictions are analogous to those of Fig. 2, i.e., for a specific center/surgeon combination. Then, we compute standardized survival probabilities for this specific center, marginalizing over the surgeons; this uses the estimator introduced in Eq. 15. Standardized survival probabilities and contrasts (versus the theoretical average) are plotted in Fig. 4: the (marginal over surgeons) predictions can be interpreted as the standardized survival probability if the entire study population was treated at the best center, while the predictions for a certain center/surgeon combination have the usual interpretation that was previously discussed. As expected, the center-specific predictions (net of surgeons) are approximately the average of the fully conditional (on centers and surgeons) predictions for the same center.

**Fig. 5:**
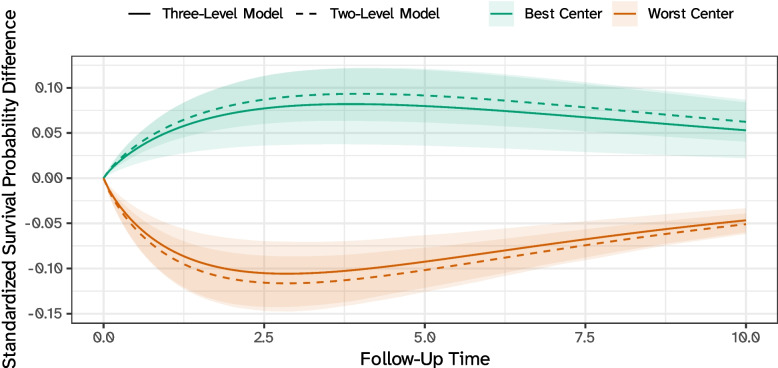
Standardized survival probability differences for the best and worst center compared to an average center, according to a three-level (solid line) and a comparable two-level (dashed line) hierarchical survival model

Center-specific predictions from the three-level model, marginalizing over surgeons, can also be directly compared, e.g., versus an average center, as in Fig. 5: once again, these comparisons are fair since we standardize over the same covariate distribution and marginalize over the same distribution of surgeon-level effects. Interestingly, one could fit a two-level model to the same data (with patients nested within centers only) and obtain predictions that are denoted by dashed lines in Fig. 5: as expected, these predictions are close. Nonetheless, fitting a three-level model provides more flexibility as one can compute more detailed predictions but also marginalize over certain dimensions whenever needed. Moreover, a three-level model can be used to partition the unobserved heterogeneity into, e.g., what is due to surgeons and what is due to centers; formal procedures are proposed elsewhere [18, 69, 70].

**Fig. 6:**
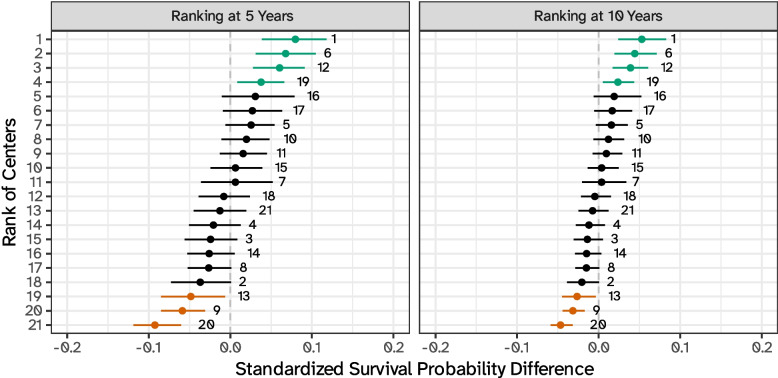
Example of centers ranking based on comparing standardized survival probabilities (marginal over surgeons) versus the theoretical average. Low-, medium-, and high-risk centers are highlighted in green, black, and red, respectively, and each data point is labeled with the unique identifier of each center

Finally, the standardized predictions produced above could be, in principle, categorized to improve reporting by defining, e.g., low-, medium-, and high-risk clusters. An example is included in Fig. 6, using the survival probability for the theoretical average center as the target value; this categorization is however subjective, to some extent, and a detailed discussion is beyond the scope of this work.

## Discussion

Throughout this manuscript we have introduced and discussed methodology to fairly compare and benchmark hierarchical units after fitting multilevel survival models. Traditionally, mixed-effects survival models have been used to accommodate the correlation between subjects belonging to the same cluster, or to account for unobserved heterogeneity: with this work, we focus on quantifying the latent performance of hierarchical units while standardizing over the observed covariates. To this end, we combined posterior predictions of the random effects with regression standardization to predict standardized survival probabilities over time. These probabilities can be compared fairly as we standardize over a common population, e.g., that of the entire study cohort. Moreover, contrasts of standardized survival probabilities can have a causal interpretation under usual assumptions. The main estimator that we introduced is close to the standardized hospital outcome rate introduced by Roessler et al. [71], which they show to be superior to the widely used standardized mortality ratio in a variety of scenarios via Monte Carlo simulation. Compared to existing approaches based on dichotomizing survival outcomes (e.g., to obtain standardized mortality ratios), the methodology discussed in this manuscript fully accommodates the time component using survival analysis methods, yielding predictions of probabilities over time and not only at a pre-specified time point. Moreover, the random effects approach is a natural choice (compared to the fixed effects approach) for generalizing results beyond the observed sample to a large population.

This approach is fully flexible and general in nature: we focus on settings with up to three levels of nesting, e.g., with patients nested within surgeons and hospitals, but any number of nesting levels can be accommodated, in principle, by adjusting the estimands (and contrasts thereof) accordingly. For instance, this could be useful in the settings of individual patient data meta-analysis, where study participants are nested within studies, as discussed by Hautekiet et al. [72]. Standardization to different subsets of the study population, such as that of the best hospital, is possible and implemented in the provided software; interpretation of the resulting estimands is closer to that of an average treatment effect on the treated [73]. Further to that, we focused on proportional hazards models, but any model that could accommodate random effects and be used to predict survival probabilities is allowed, in principle. Non-linear and time-dependent effects could easily be included as well, as long as software allows it.

To use this methodology, we need to rely on a set of assumptions. First, all usual assumptions related to survival analysis need to hold, e.g., regarding proportional hazards (if assuming so) and the shape of the baseline hazard; nonetheless, more complex and flexible models can be incorporated, e.g., via the -stmixed- estimation command in Stata [47]. The assumptions of random effects models (such as exchangeability of the clusters and normality of posterior predictions) need to hold as well. Second, standardized survival probabilities are only comparable in terms of the factors that we standardize over, and the interpretation of standardized survival probability differences as causal risk differences relies on the assumption that adjusting for the fixed effects is sufficient to control for confounding, together with all remaining assumptions required to identify a causal effect. Third, fair comparisons and benchmarking require assuming that the underlying multilevel survival model is correctly specified; while investigating this issue is beyond the scope of this paper, the literature provides promising strategies to mitigate the risk of model misspecification, e.g., via generalized additive mixed models [74]. Fourth, we rely on mixed effects models and posterior predictions of the random effects to quantify the performance of each hierarchical unit. Posterior predictions are affected by shrinkage to the population mean, with the amount of shrinkage inversely proportional to the amount of data in each cluster; this is less of an issue with large sample sizes, such as in our motivating example, but it should still be assessed on a case-by-case basis [44]. Fully-parametrized fixed effects models, penalized models, and marginal models do not need to rely on the prediction of random effects, but have significant drawbacks that were previously discussed. Fifth, we need to assume that lower-level units could, in principle, be treated at any of the higher-level units; if that was not the case, we would risk extrapolating, e.g., the effect of hospitals on patients that the hospital would not otherwise treat. Finally, Varewyck et al. [45] discuss the issue of hospital-patient interactions (e.g., interaction between lower- and higher-level units); they showed that ignoring such interactions usually has a minor impact on benchmarking the different hierarchical units, unless, e.g., some centers perform substantially better on a specific group of patients and there is strong confounding through the corresponding patient characteristic. If so, analysts may consider including such interaction effects in the multilevel model.

Extensions to this methodology could further enhance its flexibility and enable its use in more applied settings. For instance, the current software implementation only supports parametric proportional hazards hierarchical survival models fitted using the -mestreg- command in Stata; the extension to other estimation commands (or software packages) is straightforward. The contrasts between hierarchical units introduced in this manuscript rely on the proportional hazard assumption, as previously discussed. We plan to relax this assumption in future work by extending the methodology to allow for time-dependent surgeon- and hospital-level effects, e.g., within the dynamic frailty framework [75, 76]. Moreover, the focus of this manuscript is on estimation rather than testing. The proposed approach could be extended to be framed within a testing framework for detecting unusual performance, e.g., that of Ohlssen et al. [77]. Throughout this work, we have assumed nested random effects; the extension to the settings of crossed random effects is possible, but estimation may be prohibitively intensive even for a small number of levels. Thus, Stata’s -mestreg- defaults to the Laplacian approximation when a model with crossed random effects is fit, which has been known to produce biased parameter estimates, especially for the variance components: this is a substantial limitation in our settings, when focus is on the clusters and on posterior predictions of the random effects. A comparison of nested and crossed random effects is included in Schielzeth and Nakagawa [78]. We only consider terminal survival events, such as death; the extension to the settings of competing events is possible, by modeling cumulative incidence functions instead [79, 80]. Varewyck et al. [17] proposed a doubly-robust approach combining regression standardization with propensity score weighting to reduce the risk of extrapolation and model misspecification: this doubly robustness property is attractive as it offers partial protection against, e.g., the omission of important interaction terms (say, between centers and patients). Nonetheless, extending their approach to the settings of multilevel survival models is not straightforward, as combining propensity score weighting with an adjusted proportional hazards model does not necessarily yield doubly robust estimators when away from the null [81]. Alternative approaches could be used if the goal was to estimate the causal effect of measurable surgeon- or hospital-level covariates (such as operative volume), such as target trial emulation using observational data or approaches based on generalized additive mixed models [82–84]. Finally, we only considered a frequentist approach for the estimation of hierarchical survival models; extensions to the Bayesian framework are possible.

## Conclusions

We introduced and discussed an analytical approach that can be used to quantify and fairly compare the differences between hierarchical units using easily interpretable measures such as (standardized) survival probabilities and contrasts thereof. These post-estimation predictions can be computed at different points in time, with standardization performed to (possibly) different populations of interest, such as the entire study population or the population of the cluster with the best outcomes; therefore, full flexibility is allowed to tailor the analysis to several possible research questions. Interestingly, under certain assumptions, these comparisons of standardized survival probabilities can have a causal interpretation as risk differences. We provide easy-to-use software in both Stata and R to support the use of this methodology in practice, together with annotated statistical code to replicate the analyses reported and discussed in this manuscript.

## Supplementary Information

Supplementary Material 1.

## Data Availability

The synthetic dataset analyzed in this manuscript can be downloaded from the following GitHub repository, alongside all code that was used to generate it: https://github.com/RedDoorAnalytics/multilevel-survival-regstd. The repository includes annotated statistical code to fully replicate the analyses reported in this manuscript as well.
