# Supplementary material for: Standardized survival probabilities and contrasts between hierarchical units in multilevel survival models
Source: BMC Med Res Methodol. 2026 Feb 4;26:45. doi: 10.1186/s12874-026-02782-8 (PMC12931028; doi:10.1186/s12874-026-02782-8)
Supplement: Supplementary file 1 — Supplementary Material 1. [file 12874_2026_2782_MOESM1_ESM.pdf]

Supplementary Material: Standardized Survival Probabilities and Contrasts Between Hierarchical Units in Multilevel Survival Models

Authors:

Alessandro Gasparini[1], Michael J. Crowther[1], Justin M. Schaffer[2]

1. Red Door Analytics AB, Stockholm, Sweden
2. Department of Cardiothoracic Surgery, Baylor Scott & White The Heart Hospital, Plano, Texas, USA

Correspondence to: Alessandro Gasparini, alessandro.gasparini@reddooranalytics.se.

A Description and Comparison of Two-Level and Three-Level Data

The number of patients, surgeons (for the three-level data only), and centers are summarized in Table A1, with descriptive statistics for the fixed effects in Table A2. We also summarize the number of events, the total time at risk (in person-years), and the rate of events in Table A3. As expected, all summary statistics for the two datasets are close to each other, highlighting the quality of the synthetic data.

The two datasets can be downloaded, alongside annotated code in R that was used to generate the synthetic three-level dataset, from the following GitHub repository: https://github.com/RedDoorAnalytics/multilevel-survival-regstd.

Table A1: Number of patients, centers, regions in the two study datasets.

Dataset	Patients	Surgeons	Centers
Two-Level	396	—	21
Three-Level	14,604	398	21

Table A2: Summary statistics for fixed effects from the two-level and three-level datasets.

Variable	Two-Level, N = 396	Three-Level, N = 14,604
Chemotherapy	330 (83%)	12,206 (84%)
Age (> 65 Years)	213 (54%)	7,928 (54%)
Sex (Female)	66 (17%)	2,373 (16%)

Table A3: Outcome summaries for the two-level and three-level datasets. Total time at risk is quantified in terms of person-years, and event rates are expressed per 1,000 person-years.

Dataset	Sample Size	N. of Events	Total Time at Risk	Event Rate
Two-Level	396	281	987.79	284.47
Three-Level	14,604	13,862	52,418.09	264.45

B Analysis of Two-Level Data

The goal of this application is to quantify differences between centers and their heterogeneity using the two-level dataset of bladder cancer patients [Sylvester et al., 2006, Do Ha et al., 2019].

To this end, we fit a hierarchical survival model with a random center-level random intercept. We assume a proportional hazards parametrization with a Weibull baseline hazard function, adjusting for age, sex, and use of chemotherapy as fixed effects. This model is estimated using the Stata command -`mestreg`- and its default settings. For the post-estimation predictions, we use the algorithms introduced in the main body of the manuscript with 1,000 bootstrap repetitions and the percentile method to obtain confidence intervals.

Data and annotated statistical code (in Stata and R) to fully replicate these analyses can be downloaded from GitHub.

Traditionally, these have been quantified using either the estimated variance of the random intercept or the median hazard ratio (MHR). The estimated center-level variance in these settings was 0.040 (0.006, 0.284), with a corresponding MHR comparing a high-risk center versus a low-risk center of 1.209. This highlights that the hazard in high-risk hospitals is approximately 20.9% higher than the hazard in low-risk hospitals (at a median level, if we were performing all possible comparisons).

Nonetheless, the MHR is a relative measure with only an overall, population-level interpretation: we now move beyond that by estimating and presenting standardized survival probabilities for specific centers (in principle, all). Note that these quantities are directly comparable as we standardize over the same covariates distribution (i.e., that of the entire study dataset across all centers).

We start by computing posterior predictions of the random effects for each center according to the fitted model; then, we calculate standardized survival probabilities for the best and worst center (smallest and largest posterior prediction value, respectively) and a hypothetical average center (fixing a random effect to zero). These predictions are plotted in Figure B1, with contrasts of standardized survival probabilities in Figure B2. For instance, the survival probability difference between the best center and a theoretical

average center is 0.055 at 1 year, 0.085 at 5 years, and 0.055 at 10 years; conversely, the difference between the worst and average center is -0.036, -0.046, -0.024 at 1, 5, and 10 years, respectively.

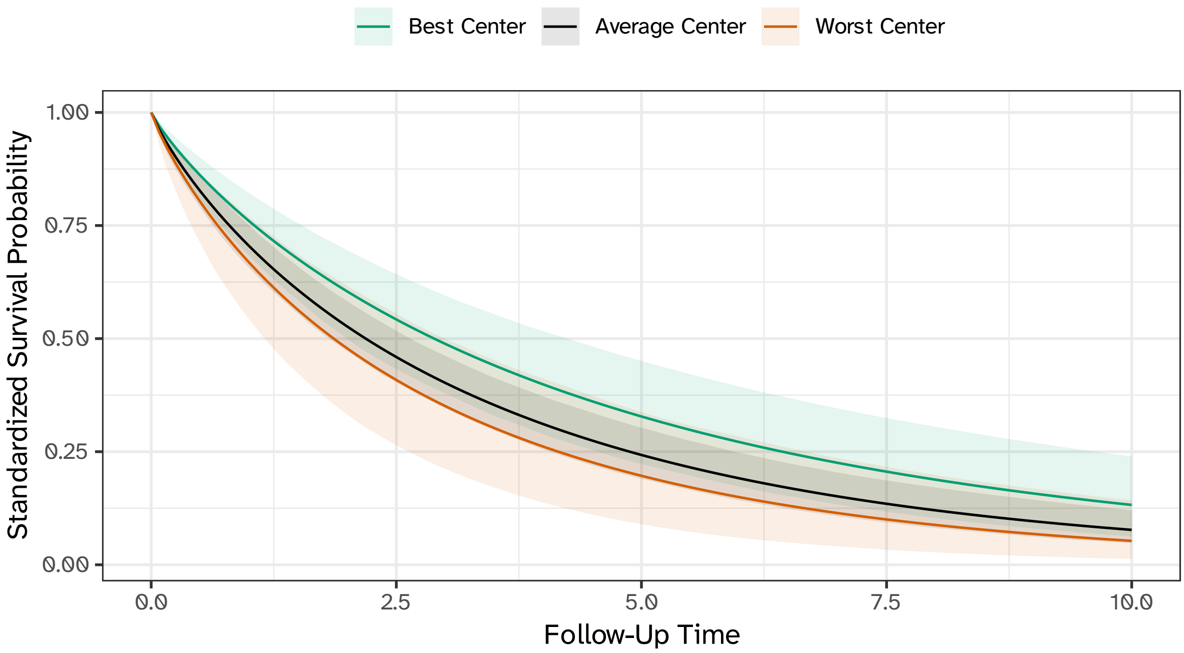

Figure B1: Standardized survival probabilities for the best, worst, and average center, according to the two-level model with patients nested within centers.

Panel A of Figure B2 assumes a theoretical average center as the reference center: we can change the reference by, for instance, taking the best center as the reference, as depicted in panel B of Figure B2. By changing the reference center we can quantify that the difference in standardized survival probability between the worst and best center is -0.092, -0.131, -0.079 at 1, 5, 10 years, respectively. Other comparisons are possible, depending on the specific research question.

These post-estimation predictions fix the posterior prediction of the random intercept for a certain center and apply that to the entire study cohort. Alternatively, one could define contrasts across the estimated distribution of the random intercept (e.g., the distribution of the latent performance of all centers, assumed to have a mean of zero and a certain variance that is estimated from data in multilevel, hierarchical survival models) by comparing, for instance, hospitals that are 1 or 2 standard deviations away from the mean. Standardized survival probabilities (and contrasts thereof) are depicted in Figure B3. Compared to the previous example of fixing the random effects to values corresponding to the best

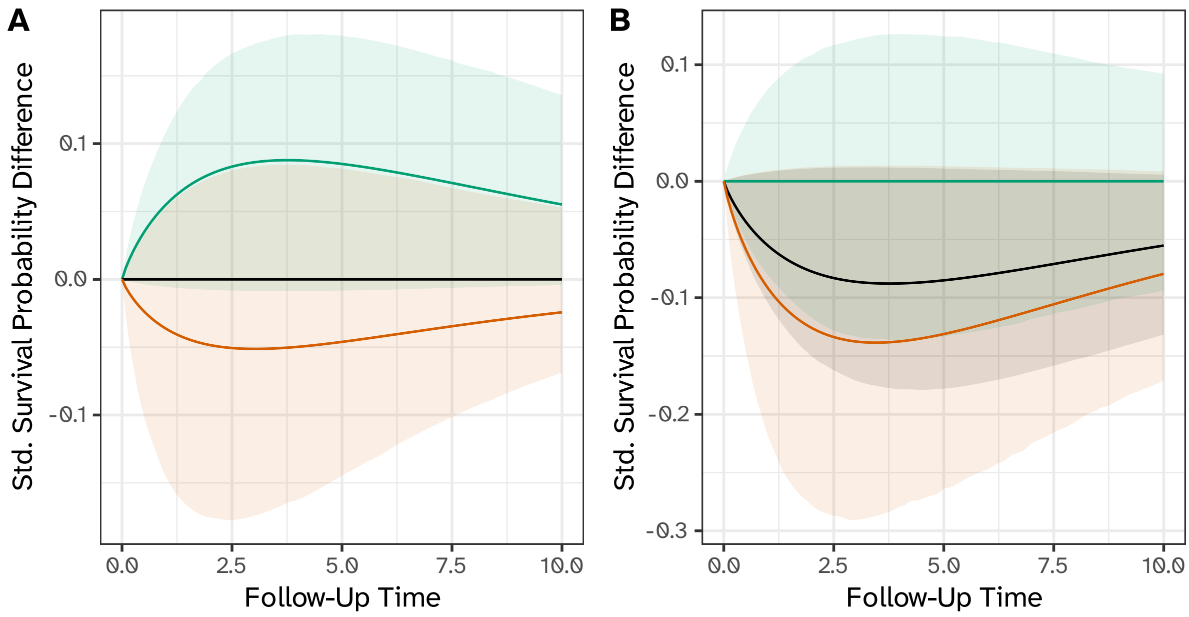

Figure B2: Standardized survival probability differences for the best, worst, and average center, according to the two-level model with patients nested within centers and assuming the theoretical average center (panel A) or the best center (panel B) as the reference.

(or worst) center, these predictions have a theoretical interpretation as we do not fix the performance of a certain hierarchical unit per se; nonetheless, these can be interesting to quantify the difference across the entire distribution of hierarchical units.

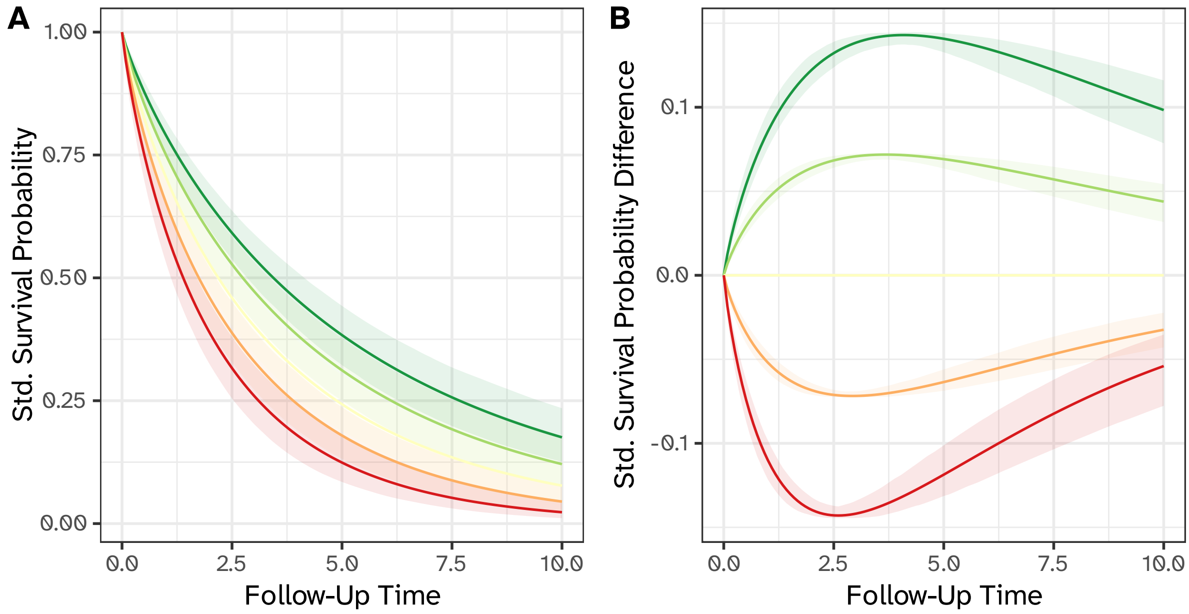

Figure B3: Standardized survival probabilities (panel A) and differences (panel B) for centers at the theoretical mean of the distribution or 1, 2 standard deviations away from it, according to the two-level model with patients nested within centers. Standardized survival differences assume the average center as the reference.

References

R. J. Sylvester, A. P. M. Van Der Meijden, W. Oosterlinck, J. A. Witjes, C. Bouffioux, L. Denis, D. W. W. Newling, and K. Kurth. Predicting recurrence and progression in individual patients with stage Ta T1 bladder cancer using EORTC risk tables: A combined analysis of 2596 patients from seven EORTC trials. *European Urology*, 49(3):466–477, 2006. doi: 10.1016/j.eururo.2005.12.031.

I. Do Ha, M Noh, J. Kim Kim, and Y. Lee. *`frailtyHL`: Frailty Models via Hierarchical Likelihood*, 2019. R package version 2.3.
